# NIV Is not Adequate for High Intensity Endurance Exercise in COPD

**DOI:** 10.3390/jcm9041054

**Published:** 2020-04-08

**Authors:** Tristan Bonnevie, Francis-Edouard Gravier, Emeline Fresnel, Adrien Kerfourn, Clément Medrinal, Guillaume Prieur, Yann Combret, Jean-François Muir, Antoine Cuvelier, David Debeaumont, Gregory Reychler, Maxime Patout, Catherine Viacroze

**Affiliations:** 1 Normandie University, UNIROUEN, UPRES EA 3830, Rouen university hospital, Haute Normandie Research and Biomedical Innovation, 76000 Rouen, France; kine@adir-hautenormandie.com (F.-E.G.); medrinal.clement.mk@gmail.com (C.M.); gprieur.kine@gmail.com (G.P.); jf.muir@adir-hautenormandie.com (J.-F.M.); antoine.cuvelier@chu-rouen.fr (A.C.); maxime.patout@chu-rouen.fr (M.P.); 2 ADIR Association, Rouen University Hospital, 76000 Rouen, France; 3 KerNel Biomedical, 76000 Rouen, France; emeline.fresnel@gmail.com (E.F.); adrien.kerfourn@gmail.com (A.K.); 4 Intensive Care Unit Department, Le Havre Hospital, 76600 Le Havre, France; 5 Institute of Experimental and Clinical Research (IREC), Pole of Pulmonology, ORL and Dermatology, Catholic University of Louvain, 1000 Brussels, Belgium; yann.combret@gmail.com; 6 Physiotherapy Department, Le Havre Hospital, 76600 Le Havre, France; 7 Pulmonary, Thoracic Oncology and Respiratory Intensive Care Department, Rouen University Hospital, 76000 Rouen, France; catherine.viacroze@chu-rouen.fr; 8 Department of Respiratory and Exercise Physiology and CIC-CRB 1404, Rouen University Hospital, 76000 Rouen, France; david.debeaumont@chu-rouen.fr; 9 Pneumology Department, Saint-Luc University Clinics, 1000 Brussels, Belgium; gregory.reychler@uclouvain.be

**Keywords:** pulmonary disease, chronic obstructive pulmonary disease, noninvasive ventilation, exercise, patient-ventilator asynchronies

## Abstract

Noninvasive ventilation (NIV) during exercise has been suggested to sustain higher training intensity but the type of NIV interface, patient-ventilator asynchronies (PVA) or technological limitation of the ventilator may interfere with exercise. We assessed whether these parameters affect endurance exercise capacity in severe COPD patients. In total, 21 patients with severe COPD not eligible to home NIV performed three constant workload tests. The first test was carried out on spontaneous breathing (SB) and the following ones with NIV and a nasal or oronasal mask in a randomized order. PVA and indicators of ventilator performance were assessed through a comprehensive analysis of the flow pressure tracing raw data from the ventilator. The time limit was significantly reduced with both masks (406 s (197–666), 240 s (131–385) and 189 s (115–545), *p* < 0.01 for tests in SB, with oronasal and nasal mask, respectively). There were few PVA with an oronasal mask (median: 3.4% (1.7–5.2)) but the ventilator reached its maximal generating capacity (median flowmax: 208.0 L/s (189.5–224.8) while inspiratory pressure dropped throughout exercise (from 10.1 (9.4–11.4) to 8.8 cmH2O (8.6–10.8), *p* < 0.01). PVA were more frequent with nasal mask (median: 12.8% (3.2–31.6), *p* < 0.01). Particularly, the proportion of patients with ineffective efforts > 10% was significantly higher with nasal interface (0% versus 33.3%, *p* < 0.01). NIV did not effectively improve endurance capacity in COPD patients not acclimated to home NIV. This was due to a technological limitation of the ventilator for the oronasal mask and the consequence either of an insufficient pressure support or a technological limitation for the nasal mask.

## 1. Introduction

Chronic obstructive pulmonary disease (COPD) is a major cause of disability and mortality worldwide [1]. Pulmonary rehabilitation (PR) has been proposed to manage its systemic effects and effectively increases functional capacity and quality of life (QoL) [2]. On the other hand, home-based noninvasive ventilation (NIV) effectively improves outcomes for those who experience diurnal hypercapnia (>55 mmHg) treated with high inspiratory pressure and rather high backup rate [3,4]. Nighttime NIV accompanying daytime PR has been suggested for these patients to improve general fatigue, gas exchanges and the benefits of PR [5,6]. In the context of a training session during PR, NIV has initially been studied during exercise, whether hypercapnia is present or not, as suggested by a recent systematic review [7] and an expert review [8]. The rationale for using NIV during exercise is based on physiologic studies supporting high intensity training [8,9]. As this might not be tolerable for most not hypercapnic patients [9], NIV is thought to relieve the work of breathing [10] and dyspnea [11]. NIV may also contribute to the redistribution of cardiac output from the respiratory muscles toward the exercising lower limb muscles, decreasing their fatigue [11], helping patients to sustain higher training stimuli, further improving the benefits of PR [7,12]. However, this notion has recently been questioned by Anekwe et al. who found that both patient-ventilator asynchrony and ventilator technological limitation may occur when patients reach high intensity during an incremental cardiopulmonary exercise testing [13]. As this test does not represent the usual training modality used during PR [14,15], their occurrence during a constant high intensity endurance exercise testing deserves to be studied. Moreover, the influence of the interface used remain unknown. Nasal or oronasal masks (NM and ONM, respectively) are used inconsistently across studies [16,17] and many patients stop training due to interface discomfort [16,18]. The choice of the interface is of real concern because the high level of ventilation during exercise necessitates breathing through the mouth, which in turn may elicit important leaks and patient-ventilatory asynchrony that would compromise exercise capacity, therefore requiring an ONM. On the other hand, ONM is usually perceived as less comfortable than NM and could reduce the compliance [8].

The aim of this study was to assess whether the type of NIV interface affects endurance capacity in COPD patients who were not eligible for home NIV and to describe the incidence, the type and the influence of patient-ventilator asynchronies on endurance capacity according to the interface. The secondary objectives were to evaluate the effects of interface type on perceived exertion, comfort, cardiopulmonary parameters and to assess the ventilator capacity to deliver sufficient support using breathing pattern, flow and pressure tracing analysis as an indirect surrogate for ventilator performance. It was hypothesized that, due to the mode of breathing during exercise (i.e., predominantly oral), high levels of leaks and patient-ventilator asynchrony would occur with nasal masks and would compromise exercise endurance.

## 2. Experimental Section

### 2.1. Study Design and Participants

This prospective, randomized cross-over trial was approved by the French ethics committee Nord-Ouest I (CPP-SC 010/2015). It was prospectively registered at https://clinicaltrials.gov (NCT02796599) and is reported according to the CONSORT statement.

All consecutive patients with clinically stable (one month) severe to very severe COPD and a ventilatory limitation during exercise referred for PR at Aide à Domicile pour les Insuffisants Respiratoires (ADIR) Association, Rouen University Hospital, France, were screened for eligibility between June 2016 and February 2018. They were not included if they were eligible for long-term NIV [3]. Details about inclusion, non-inclusion and exclusion criteria are available in Appendix A. Written informed consent was obtained from all patients.

### 2.2. Clinical and Functional Evaluation

As part of their baseline assessment which took place within the two weeks before attending the PR program, all patients underwent a complete evaluation including pulmonary function tests and evaluation of exercise capacity using the six-minute walk test and CPET on the same day. Subsequently, patients were offered to participate in the study. Details about the procedures are available in Appendix B.

### 2.3. Protocol

Those patients who accepted to participate in the study took part in three visits. These visits were separated by a minimum of 48 h and took place within a maximum of two weeks. The experimental procedure was successfully respected for each of the participants.

Visit 1: First, patients were allowed 15 to 20 min to become accustomed to the NIV (Trilogy, Respironics Inc., Murrysville, PA, USA) at rest with both NM and ONM (Eson TM and Simplus TM, Fisher & Paykel Healthcare, Auckland, New-Zealand) in a sitting position. NIV was delivered with a single limb circuit and both masks were provided with intentional leaks port. NIV settings at rest were positive expiratory pressure (PEP) 4 cmH2O, pressure support 5 cmH2O.

Secondly, patients performed a constant workload exercise testing (CWET) on spontaneous breathing (SB) which was used as an anchor of perceived exertion for the subsequent titration of the NIV parameters.

Third, after a 15-min resting period, another exercise session was carried out at the same workload intensity as the CWET in SB in order to determine the appropriate NIV settings for visits two and three. Patients were asked to breathe through the nose as long as possible when using NM. Based on previous studies suggesting that higher pressure would provide more benefits in exercise capacity, dyspnea and respiratory work of breathing [19,20,21,22], and that the addition of a PEP could further improve these benefits (counterbalancing for the intrinsic PEP) [22,23], the aim of the titration was to reach both the highest pressure support and PEP tolerated. Both interfaces were tested according to the randomization and the following protocol was used: first, progressive rise in pressure support (2 cmH2O/minute) and secondly PEP (1 cmH2O/min; using the pressure support level previously chosen) as long as the patient felt more comfortable compared with the first CWET during SB or until it became uncomfortable. The highest pressure support and PEP tolerated were used for the subsequent evaluations. Thirdly, pressure rise time was also adjusted according to the patient’s tolerance. “Auto-Trak” mode was used for the inspiratory and expiratory trigger.

Visits two and three: Patients performed a CWET under NIV with NM or ONM (randomized order) using the settings determined during visit one.

CWET: CWET were performed according to current guidelines [24]. After a one-minute warm-up period (unloaded), patients were asked to maintain a load corresponding to 75% of Wmax at 70 revolutions per minute (rpm) until exhaustion. No encouragement was given except the time every minute. The test ended when patients stopped because of symptoms or when the cycling speed dropped by 10 rpm for more than 10 s.

### 2.4. Randomization

The randomization was carried out using a computer-generated sequence (www.randomized.org). After completion of the first CWET with SB, the order of the two subsequent tests (with NM or ONM) was randomized by an individual unrelated to the study (concealed allocation).

### 2.5. Outcomes

The primary outcome was maximal endurance time (Tlim) of the CWET between the three conditions (SB, NM and ONM).

Secondary outcomes: a comprehensive analysis of the flow and pressure raw data from the ventilator were used to assess patient-ventilator asynchrony, breathing pattern, and were used as a surrogate for ventilator performance. Further methodological details are provided in Appendix C.

Interface comfort: mask comfort was assessed after both NIV tests using a visual analogue scale (VAS), ranging from 0 (extremely uncomfortable) to 10 (extremely comfortable).

Perceived exertion: dyspnea and lower limb fatigue were both assessed at rest and every 30 s using the Borg scale [25].

Transcutaneous oxygen and carbon-dioxide measurement: transcutaneous oxygen saturation (SpO2) and transcutaneous carbon-dioxide partial pressure (TcPCO2) were continuously recorded using a capnograph (SenTec, ResMed, San Diego, CA, USA) at the earlobe. It was set up at least 20 min before each CWET to allow calibration of the signal. In order to assess SpO2 and TcPCO2 signals at a similar time point, TcPCO2 was analyzed with a 2 min lag-time [26].

### 2.6. Statistical Analysis

A sample size calculation was carried out to detect a clinical positive effect of NIV using an ONM compared with NIV using a NM on endurance exercise capacity during a constant CWET (assessed as Tlim (s)). Accordingly, 15 patients were required to detect a minimal clinical important difference of 101 s (SD 100 s) in Tlim [27] with a 95% power at the 0.05 significance level. We planned to recruit 21 patients to account for attrition due to intolerance of NIV in people not eligible to and to further improve the power of the study (99% power in the situation where all patients would complete the study).

Normality of the distribution of each variable was assessed using a Shapiro–Wilk test. Categorical data were expressed as counts (%) and continuous data were expressed as means (SD or 95% CI) or medians (25th–75th percentiles) depending on the distribution.

Cardiorespiratory outcomes were analyzed both at iso time and at Tlim. Iso time was defined as the Tlim of the shortest CWET. Comparisons between interfaces were performed using a paired t-test or a Wilcoxon Signed Rank test. Multiple comparisons were performed using paired repeated measures of analysis of variance (ANOVA) or Friedman tests. In the case of a significant difference, Wilcoxon tests were performed to explore pairwise comparisons and a Bonferroni correction was applied. Relationships were assessed using Pearson or Spearman correlation tests. Patients with an increase in Tlim for more than 101 s or 33% with any interface compared with the SB test were deemed as improvers [27] and were considered for further analysis in order to assess whether baseline characteristics might predict responsiveness to NIV using an independent t-test or a Mann-Whitney test according to the data distribution.

In order to assess changes in respiratory parameters and ventilator performance, a comparison was made between the cycle by cycle mean of the 60 first and the 60 last seconds of CWET. Patients with less than two minutes of records were excluded for this analysis (four with ONM and six with NM. Moreover, the variation of each indicator between the beginning and the end of exercise was calculated as follows: Indicatorvar = Indicatorend − Indicatorbeg.

Comparison in the proportion patient with AI > 10% between interfaces was performed using the Fisher test.

## 3. Results

### 3.1. Patients

**Figure 1:**
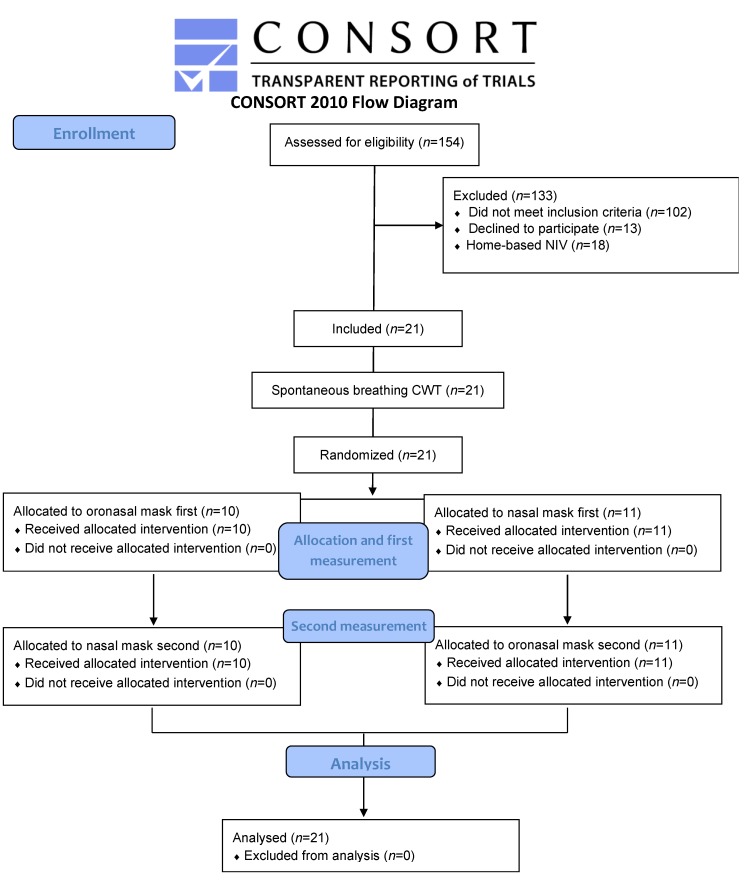
Flow-chart. NIV: noninvasive ventilation; CWET: constant workload exercise testing.

One hundred fifty-four patients were screened for eligibility and twenty-one were included in the study. There were no dropouts (Figure 1).

Patients characteristics are shown in Table 1. Nine (43%) were female and five (24%) were long term oxygen users. All had severe obstruction (mean FEV1%: 35.3% (±8.3)), were severely hyperinflated (mean RV/TLC: 0.6 (±0.1) and had impaired exercise capacity (mean VO2peak: 12.1 mL/kg/min (±2.8).

NIV Parameters: See Table S1.

### 3.2. Primary Outcome

**Figure 2:**
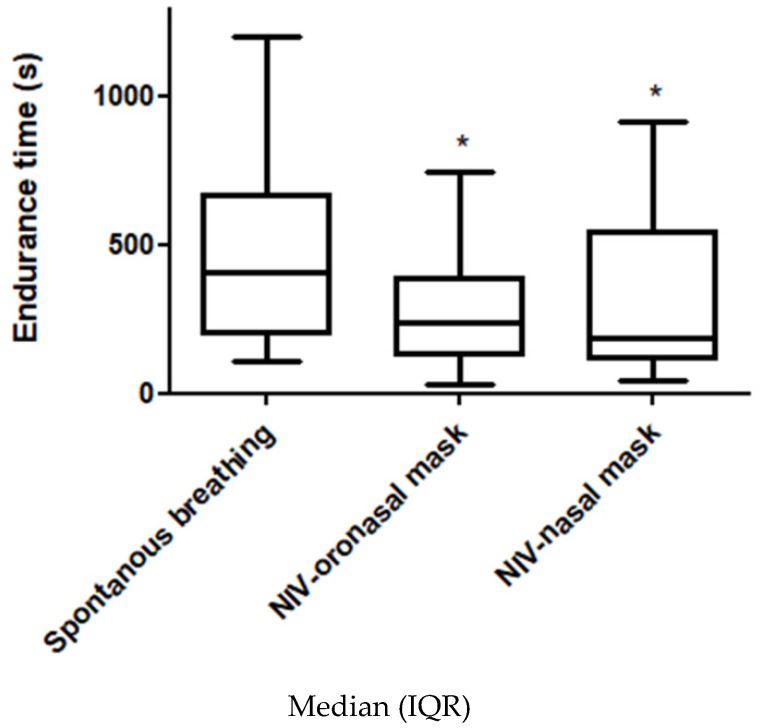
Endurance time for each condition. NIV: noninvasive ventilation. *: significantly lower than the spontaneous breathing condition, *p* < 0.02 (Friedman one-way analysis of variance and Wilcoxon post hoc analysis).

There were no order effects between tests (*p* = 0.84). There was a significant difference in Tlim between SB, ONM and NM due to the significant reduction in this variable with both masks (respectively 406 s (IQR 197–666), 240 s (IQR 131–385) and 189 s (IQR 115–545), *p* < 0.01). However, there was no significant difference between interfaces (*p* = 0.34) (Figure 2).

Three patients (14%) were considered as improvers and eighteen (86%) as non-improvers. Only inspiratory capacity (IC%) was significantly higher in improvers (81% (SD 1) versus 62% (SD 17), *p* < 0.01). There was no significant difference in the proportion of improvers among patients whose TcPCO2 increased for more than 4 mmHg during the SB test compared with those whose TcPCO2 did not increase.

### 3.3. Secondary Outcomes

#### 3.3.1. Patient-Ventilator Asynchrony

Data from 42 exercise sessions with NIV (for a total of 13,415 s and 5136 respiratory cycles) were analyzed. Total asynchrony index (AI) (%) according to the interface is shown in Table 2.

The proportion of patients with AI > 10% for ineffective efforts (IE) was significantly higher with NM than with ONM (33.3 versus 0%, *p* < 0.01). The difference was not significant regarding double-triggering (DT) (19 versus 0%, *p* > 0.1) or auto-triggering (AT) (5 versus 0%, *p* = 1). During tests with NM, endurance time was significantly higher in subjects with more than 10% IE AI (592.9 s (SD 385.3)) compared with those with 10% or less (258.9 s (SD 134.3)), *p* < 0.05).

The median total major AI (%) significantly increased only for NM from the beginning (5% (IQR 0–13.6) to the end of exercise (15% (IQR 3.6–45), *p* = 0.04) mainly due to a significant increase in IE (from 0% (IQR 0–4.6) to 2.9% (IQR 0–30), *p* < 0.05) (Figure S1).

There was no significant relation between NPD, total major asynchronies AI% and both endurance time or interface comfort for the two interfaces.

#### 3.3.2. Flow and Pressure Tracing Analysis

Comparison in flow and pressure measurement between the beginning and the end of exercise and between interfaces are shown in Table S2.

Comparison of flow and pressure between subjects with IE AI ≤ 10% or > 10% for NM are shown in Table S3.

#### 3.3.3. Cardiopulmonary Outcomes

Compared with SB, SpO2 increased with both ONM and NM (respectively 95.2% (SD 1.6), 97.1% (SD 1.1) and 97% (SD 1.2), *p* < 0.05) at rest. Conversely, TcPCO2 was significantly reduced with the ONM compared with SB (36 mmHg (SD 3) and 38 mmHg (SD 3.6) respectively, *p* < 0.05). Vt, unintentional leaks and RR were not significantly different at rest between interfaces while they significantly differed during exercise. Table 3.

Other respiratory parameters are shown in Table S4.

#### 3.3.4. Interface Comfort

There was no significant difference in comfort between the ONM and NM (respectively, 4.95 (SD 2.5) and 4.86 (SD 2.6), *p* = 0.88).

#### 3.3.5. Perceived Exertion

There were no significant differences between the three tests for dyspnea or lower limb fatigue at iso time and for dyspnea at Tlim. Compared with SB, lower limb fatigue was significantly reduced with both ONM and NM (respectively 6.3 (SD 2.9), 4.8 (SD 3.2) and 5.1 (SD 3.1), *p* < 0.05).

#### 3.3.6. Relationship between Outcomes

There was no significant relation between NPD, total major AI% and both endurance time and interface comfort for the two interfaces. Additionally, there was a positive significant relationship between IE AI% and endurance time with NM (r = 0.47, *p* = 0.03).

## 4. Discussion

The results of this study show that NIV during exercise did not improve endurance exercise capacity with any type of interfaces (an may even worsened exercise capacity), without any significant differences between them. Patient-ventilator asynchrony was relatively infrequent with ONM but significantly increased with NM (median AI: 12.8%). Particularly, IE AI% was clinically relevant for 33% of the patients with NM and was positively correlated with endurance time (r = 0.47, *p* = 0.03). The comprehensive flow and pressure tracing analysis revealed that the ventilator likely reached its performance limits, particularly with NM.

Because of its complexity and the many parameters influencing its use, NIV during exercise is a much-debated topic with divergent results, particularly when used over a course of PR. Indeed, previous acute and physiological studies has mostly demonstrated a significant positive effect of NIV on exercise capacity [11,12,28,29,30] or no positive effects [31,32], while long-term studies remain inconclusive [7,18,33]. In this context, the detrimental effect of NIV on endurance exercise capacity found in the present study was quite unexpected and it is therefore difficult to differentiate between a real worsening in endurance exercise capacity or a lack of improvement (there is also a possibility of a type 1 statistical error). Conservatively, other factors also contribute to explain the lack of improvement observed with NIV.

First, we found that those patients with a lower IC% at rest were more likely to be not-improvers. This is in line with (i) Oliveira et al., who found that NIV adversely affects “central” hemodynamics adjustments to exercise and was associated with a lack of improvement in exercise capacity in patients severely hyperinflated at rest and (ii) O’Donnell et al. who had previously shown that the inability to further expand Vt during exercise was an important factor contributing to exercise limitation in hyperinflated COPD patients [34]. Because our participants were further hyperinflated than those who participated in the study of Oliveira et al. and much more than those involved in previous studies which found positive effects of NIV [11,30], it is likely that they could not further expand their Vt even with NIV due to their hyperinflation even though they experienced the central hemodynamics side effects of NIV. On the other hand, subjects with a lower extent of hyperinflation who are still able to expand their Vt may still benefit from NIV [12].

Additionally, other factors such as the low pressure support used [3,4], NIV-induced hypercapnia [31,35] or the patient’s selection (i.e., without chronic hypercapnic respiratory failure (CHRF)) may also explain the lack of improvement in Tlim. Indeed, higher inspiratory support may have led to a positive effect of NIV as suggested by Gloeckl et al. who found a significant improvement in endurance capacity with high-pressure NIV during exercise in patients already undergoing long-term NIV for CHRF [36]. Although some other studies suggest the use of the highest tolerable inspiratory support [37], the median support was only 8 cmH2O in the present study and higher levels were not tolerated by these patients who were naive to NIV. However, it was within the ranges of those used in studies that found that NIV improved endurance capacity [19,20] and matched with the level of pressure support titrated to comfort used in Anekwe et al. [13]. Based on the available evidence, we included patients with severe obstruction and ventilatory limitation [7,8,38] and did not include those patients who were eligible to home NIV to prevent bias relating to experience. However, although the NIV was initiated at rest and then titrated during exercise, in laboratory conditions and by a physiotherapist experienced in NIV, it is possible that the patients were insufficiently acclimatized to the NIV. Moreover, these negative results in patients not eligible for long-term NIV supports two recent studies performed specifically in patients with CHRF patients [29,38]. Altogether, these results suggest that, during exercise, NIV may be particularly effective in patients who are already under home NIV and tolerate higher pressure support (i.e., CHRF).

Beyond the selection of the patients and the pressure support used, our results support other mechanisms to explain the decreased endurance performance with NIV, which differ between interfaces.

### 4.1. Oronasal Interface

There were few patient-ventilator asynchronies with ONM (median total AI < 4%) and their occurrence was not related with endurance time, so they were likely to be clinically irrelevant. Conversely, flow measurements significantly increased throughout exercise and were superior to those with NM. Particularly, Fini progressively rose up with ONM (+24 L/min compared with +3 L/min with NM, *p* < 0.01). Theoretically, it is not supposed to increase during exercise if the ventilator sufficiently assists the patient, suggesting a lack of power of the ventilator. Moreover, Fmax, mean and Fmax, end reached about 200 L/min (which is the maximal flow generating capacity of the ventilator used according to the manufacturer), while Pinspi significantly decreased all along the exercise suggesting that the ventilator was unable to maintain the set pressure despite the fact the maximal flow generating capacity was reached. In addition, the pressure rise time (τ) was set to the fastest setting available on the ventilator (i.e., “1”, corresponding to a duration of 100 ms to reach the set pressure from the beginning of the inspiratory cycle according to the manufacturer data). However, τini and the mean τ for both interfaces were largely superior this value (400 ms), suggesting that the ventilator was unable to rise the pressure as quickly as set, again suggesting a technological limitation.

These observations extend those from a previous study which also found markers of technological limitation during exercise in COPD patients, even though an intensive care unit ventilator was used [13]. This supports the idea that the ventilator was not able to maintain even the low pressure support used in the present study (although low, Pinspi decreased all along the exercise while the maximal flow generating capacity of the ventilator was reached), therefore it is unlikely that a higher pressure support could have been reached. Therefore, although potentially contributing, the low inspiratory support used was not the primary explanation for the lack of improvement in the endurance exercise capacity observed in the present study because higher pressure would have been limited by technological limitations.

### 4.2. Nasal Interface

The reason for the altered performance with NM is more complex. IE was the most frequent, clinically relevant patient-ventilator asynchrony (33% of the patients reached the clinical level of significance of 10% [39]) and was significantly related with endurance performance. Although the positive nature of this relation is primarily surprising, it can be explained by several factors. First, those patients with more than 10% IE AI had significantly more leaks and a negative value of Fini, var. This strongly suggests that these patients began to exercise breathing through the nose and then by the mouth when exercise became more difficult (Figure S1) in such a way that these patients breathed “over” the ventilator, eliciting IE and lower Vt and RR recorded by the ventilator at time limit (IE cycle values not recorded (Table 3)).

This is strengthened by the fact that AI% significantly increased with NM from the beginning to the end of exercise and that those patients with a high level of asynchrony had a significantly higher Tlim, as in the spontaneous breathing test.

On the other hand, two thirds of the patients did not open the mouth and had a similar pattern to ONM (i.e., less asynchronies but a decreased endurance capacity). Contrary to ONM, the Fmax, end (about 180 L/min) was below the maximal generating capacity of the ventilator and the set pressure support was reached, suggesting that it could have been further increased. This observation raises concern about the possibility to adjust NIV parameters throughout the exercise. This difference with ONM likely lies in the higher resistance of the upper airway which helped to reach the set pressure with a lower flow. However, higher support was not tolerated by the patients and the relatively low margin for the flow to increase (about 20/min) makes the possibility to further expand it difficult to achieve without reaching the technological limitation of the ventilator. Moreover, some markers of the technological limitations of the ventilator were already present (pressure rise time set not reached). Altogether, these results suggest that the important amount of IE observed in a third of the patients (who breathed through the mouth at the end of exercise) was a consequence either of a direct technological limitation or an insufficient support that could not have been further expanded due to technological limitation.

### 4.3. Implication for Practice and Research

The main strength of this study is that it was conducted in a condition close to that which would be used if NIV was used to sustain higher training intensity during PR (75% Wmax). Our negative results do not exclude a positive effect of NIV at a lower relative intensity [29], where the inspiratory flow is lower and may not exceed the ventilator generating capacity. Accordingly, NIV should be used as a “starter” to initiate PR and more rapidly reach the prescribed length of training (generally 30–45 min [14,40,41]) rather than a “booster” to sustain higher training intensity as suggested by physiological studies [9,42,43]. Moreover, this study supports the use of ventilator displaying Fmax value on the monitoring screen to help clinicians to assesses whether the ventilator is powerful enough to relieve respiratory effort for a given exercise. Finally, our results suggest that the respiratory support needed by the patients, as well as patient-ventilator asynchronies, varies throughout the exercise. Therefore, the effects of automated modes that could adapt more easily to the different exertions of exercise (such as those using a volume-assured pressure support and automated PEP) deserve to be studied.

### 4.4. Limits of the Study

First, neither the patients nor the assessor were blinded. However, it is unlikely to influence patient-ventilator asynchronies or the technological capacity of the ventilator. Secondly, the SB condition was carried out first, and was not randomized. This choice was made to allow a perceived exertion anchor for the subsequent NIV parameters titration because most of the patients with COPD are not used to exercise. Moreover, it helped to avoid any possible ordering effect relating to the procedure between masks. Lastly, flow pressure tracing and respiratory parameters were derived from the raw data of the built-in software of the ventilator respectively and not from an external pneumotachograph and pressure transducer, which may have introduced some errors in the measurements due to leaks [44,45] and precluded any comparison between the SB tests with both masks.

## 5. Conclusions

In patients with COPD not acclimated to long-term home mechanical ventilation, during-exercise NIV delivered through either NM or ONM does not improve endurance capacity (and may even worsen it). Patient-ventilator asynchrony was uncommon with ONM and endurance performance was likely impaired due to technological limitation of the ventilator. Patient-ventilator asynchrony, particularly IE, was more frequent with NM and reflects the fact that some patients shunted the ventilator by breathing through the mouth. This likely occurred either because of an insufficient pressure support (with few possibilities to increase it due to technological limitations) or directly due to a technological limitation of the ventilator.

## Figures and Tables

**Table 1:** Characteristics of the participants.

Variable, (Units)	Participants (*n* = 21)
Female (*n*)	9 (43) ^a^
Age (years)	58.9(10.7) ^b^
Height (cm)	170 (8.9) ^b^
Body mass (kg)	66.1 (11.9) ^b^
BMI (kg/m²)	22.8 (3) ^b^
FEV1 (L)	0.9 (0.8–1.3) ^c^
FEV1 (%)	35.3 (8.3) ^b^
FVC (L)	2.6 (0.8) ^b^
FEV1/FVC (% ratio)	40.2 (8.8) ^b^
RV (L)	4.5 (0.9) ^b^
RV (%)	216 (43) ^b^
TLC (L)	7.4 (1.3) ^b^
RV/TLC	0.6 (0.1) ^b^
IC (L)	1.7 (0.6) ^b^
IC (%)	65 (17) ^b^
VO2 peak (mL/kg/min)	12.1 (2.8) ^b^
Arterial blood gas	50 (40–70) ^c^
PaO2 (mmHg)	67.5 (12) ^b^
PaCO2 (mmHg)	38.3 (4.5) ^b^
pH	7.44 (0) ^b^
HCO3- (mmol/L)	24.8 (23–25) ^c^
Wmax (W)	50 (40–70) ^c^
6MWT (m)	413.5 (99.1) ^b^
LTO (*n*)	5 (24) ^a^
BODE	4.3 (1.7) ^b^

^a^ Values expressed as numbers (%); ^b^ Values expressed as means (SD); ^c^ Values expressed as medians (25th–75th percentile). FEV1/FVC is expressed as a percentage ratio. BMI: body mass index; FEV1: forced expiratory volume in one second; FCV: forced vital capacity; RV: residual volume; TLC: total lung capacity; IC: inspiratory capacity; VO2peak: maximal oxygen consumption; PaO2: oxygen arterial partial pressure; PaCO2: carbon dioxide arterial partial pressure; HCO3-: bicarbonates; Wmax: maximal workload achieved during cardiopulmonary exercise testing; 6MWT: six-minute walk test; LTO: long-term oxygen; BODE: Body mass index, airflow Obstructive, Dyspnea, and Exercise capacity index.

**Table 2:** Asynchrony index according to the interface.

Event (%)	Interface		Between-Group Comparison
	Oronasal Mask (*n* = 21)	Nasal Mask (*n* = 21)	*p*
Normal cycles	95.5 (91.6–97.0)	82.5 (47.1–95.5)	*p* < 0.01
Ineffective triggerings	0 (0–0.1)	2.1 (0–21.5)	*p* < 0.01
Double-triggerings	0.2 (0–1.6)	2.7 (0.7–8.6)	*p* < 0.01
Auto-triggerings	1.7 (0–3.6)	1.7 (0.4–2.5)	NS
Premature cyclings	0.2 (0.0–1.9)	0.9 (0.0–6.9)	NS
Delayed cyclings	0.0 (0.0–0.8)	0.0 (0.0–2.3)	NS
NDP	96.6 (94.8–98.3)	87.2 (68.4–96.8)	*p* < 0.01
Total major asynchrony events	3.4 (1.7–5.2)	12.8 (3.2–31.6)	*p* < 0.01

Values are expressed as medians (25th–75th percentile). NDP: sum of normal cycle, premature and delayed cycling. Total major asynchrony events are the sum of ineffective efforts, double-triggering and auto-triggering. NS, not significant.

**Table 3:** Effects of non-invasive ventilation on cardiopulmonary outcome at iso-time and time limit.

Variables, (Units)	Constant Workload Exercise Testing			Between-Group Comparison
	Spontaneous Breathing (*n* = 21)	Oronasal Mask (*n* = 21)	Nasal Mask (*n* = 21)	*p*
Iso-time				
Heart rate (bpm)	111.6 (17)	118.2 (13.4)*	116.5 (11.1)	*p* < 0.05
SpO2 (%)	93.1 (3)	93.5 (2.4)	93.4 (2.4)	NS
TcPCO2 (mmHg)	39.1 (4.4)	40.2 (3.2)	39.9 (3.9)	NS
Respiratory rate (cpm)		27.6 (6.7)	24.7 (8.6)	NS
Vt (mL)		1333.6 (486.2)	784.3 (486.2)	*p* < 0.01
Unintentional leaks (L/min)		12.2 (9.6)	28.3 (29.8)	*p* < 0.03
Time limit				
Heart rate (bpm)	121.1 (15.3)	120.5 (13.7)	120 (13.7)	NS
SpO2 (%)	92.8 (3.2)	93.1 (2.6)	93.1 (2.9)	NS
TcPCO2 (mmHg)	38.5 (4.5)	39.5 (2.9)	40 (4)	NS
Respiratory rate (cpm)		30.3 (5.4)	24.9 (9.6)	*p* < 0.01
Vt (mL)		1225.6 (494.2)	828.3 (506.2)	*p* < 0.01
Unintentional leaks (L/min)		11.7 (9.6)	28.9 (31.3)	*p* < 0.04

Values expressed as means (SD). *: significantly higher than spontaneous breathing, *p* < 0.04. Respiratory rate, Vt and unintentional leaks were recorded by the built-in software of the ventilator. bpm: beats per minute; SpO2: transcutaneous oxygen saturation; TcPCO2: transcutaneous carbon-dioxide partial pressure; cpm: cycles per minute; Vt: volume tidal; NS: not significant. NS, not significant.

## Supplementary Materials

The following are available online at https://www.mdpi.com/2077-0383/9/4/1054/s1, Figure S1: Comparison in ventilatory dynamic between the beginning (**A**) and the end (**B**) of exercise in a representative subject with nasal mask. Table S1: Noninvasive ventilation settings. Table S2: Comparison in flow and pressure between the beginning and the end of exercise. Table S3: Comparison of flow and pressure between subjects with IE AI ≤ 10% or > 10% for nasal interface. Table S4: Comparison of cycle time, instantaneous respiratory rate and Ti/Ttot between interfaces.

## Appendix A

Inclusion: non-inclusion and exclusion criteria.

Inclusion criteria:

- Stable (on month) severe to very severe COPD referred for pulmonary rehabilitation. The clinical diagnosis of COPD was based on a ratio between forced expiratory volume in one second (FEV1) and forced vital capacity (FVC) < 0.70;
- Age ≥ 18 years;
- FEV1 < 50% predicted;
- Ventilatory limitation (breathing reserve < 30%) during the initial cardiopulmonary exercise testing [7,38].

Non-inclusion criteria:

- Eligible to home noninvasive ventilation according to [3]:
- PaCO2 ≥ 51.9 mmHg and pH > 7.35 at rest;
- Potential pregnancy;
- Under guardianship;
- Refusal to consent.

Exclusion criteria:

- Acute exacerbation of COPD (according to the Anthonisen’s criteria [46]) before completion of the study.

## Appendix B

Clinical and functional evaluation.

Pulmonary function: Pulmonary function tests were performed according to the American Thoracic Society (ATS) and the European Respiratory Society (ERS) guidelines with plethysmography (Masterscreen, Jaeger, Wittsburg, Germany). Values were expressed as a percentage of established theoretical values for European population [47].

Six-minute walk test: The six-minute walk test was carried-out according to the ATS and ERS guidelines in a 30-m corridor [48,49]. The test was performed twice and the best distance was used for analysis.

Cardiopulmonary exercise testing: Cardiopulmonary exercise testing was performed on an electromagnetic braked cycloergometer (Ergoselect 200, Ergoline, Bitz, Germany). Following a 3-min warm-up period, incremental ramp exercise was applied up to exhaustion (5–20 W/min). A pneumotachograph and gas analyzer (Ergocard, Medisoft, Louvain, Belgium) were used to measure gases (oxygen consumption and carbon dioxide production breath by breath) through a face mask (Hans Rudolph, Inc., Kansas City, MO, USA). The last ramp maintained before stopping the exercise was used to determine the maximal workload (Wmax).

## Appendix C

Secondary outcomes.

A comprehensive analysis of the flow and pressure raw data from the ventilator were used to assess patient-ventilator asynchrony, breathing pattern, and were used as a surrogate for ventilator performance.

Patient-ventilator asynchrony: According to the framework proposed by the SomnoNIV group, the following asynchrony events were assessed [50,51,52,53]:

Rate asynchronies: rate asynchronies are defined as a mismatch between ventilator and patients’ rates [53] and include:

- Ineffective effort (IE): IE is characterized by an inspiratory effort not assisted by the ventilator. It can be identified as a drop of airway pressure associated with increase or decrease of airflow (if occurring during expiratory or inspiratory phase respectively) [52];
- Double-triggering (DT): DT is characterized by two mechanical cycles triggered by the patient, separated by a very short expiratory time (< 30% of mean inspiratory time) [52];
- Auto-triggering (AT): AT is characterized by the presence of mechanical cycle unrelated to patient’s spontaneous breathing [52];

Intracycle asynchronies: intracycle asynchronies were defined as a distortion of the flow and pressure curves during inspiration and/or expiration [53] and include:

- Premature cycling (PC): PC reflects a situation where the end of the mechanical insufflation anticipates patient’s own inspiration termination [52];
- Delayed cycling (DC): Otherwise to PC, DC is a condition where the mechanical insufflation exceeds the patient’s own neural expiration [52].

Quantification of asynchronies: Rate asynchronies (IE, DT and AT) were considered as major asynchrony event [52]. PC and DC were considered as minor event. Cycles corresponding to none of these categories were considered as normal. Normal cycles, PC and DC were pooled and termed as NPD.

Each major asynchrony event and total major asynchronies were standardized using the previously described asynchrony index (AI(%)) dividing the asynchronous breath by the sum of ventilator cycles and IE, expressed as percentage [39,50,51]. An AI > 10% was considered as clinically relevant [39,50,51].

Breathing pattern, flow and pressure tracing analysis: Respiratory rate (RR), tidal volume (Vt) and unintentional leaks were recorded by the built-in software of the ventilator. As the ventilator recorded total leakage, unintentional leaks were estimated at each time point by subtracting the intentional leak for each mask at a given pressure level (manufacturer’s data) from total leak. For every patient with both interfaces and for every NPD cycle, the following data were collected: cycle time, instantaneous respiratory rate (IRR; calculated as the inverse of cycle time), pressure rise time (τ), Ti/Ttot ratio, flow at the beginning of the cycle (Fini), maximal flow (Fmax), minimal pressure (Pmin), maximal pressure (Pmax) and mean inspiratory pressure (Pinspi; from the beginning of the cycle to the transition to minimal pressure).

Moreover, several indicators were calculated from cycle by cycle analysis as follow:

- Mean differential flow (Fdiff) = Fmax, mean − Fini, mean;
- Mean differential pressure (Pdiff) = Pmax, mean − Pmin, mean.
