# Supplementary material for: NIV Is not Adequate for High Intensity Endurance Exercise in COPD
Source: J Clin Med. 2020 Apr 8;9(4):1054. doi: 10.3390/jcm9041054 (PMC7230463; doi:10.3390/jcm9041054)
Supplement: Supplementary file 1 [file jcm-09-01054-s001.pdf]

J. Clin. Med. **2020**, *9*, 1054; 10.3390/jcm9041054

Supplementary Materials: NIV Is not Adequate for High Intensity Endurance Exercise in COPD

Tristan Bonnevie *, Francis-Edouard Gravier, Emeline Fresnel, Adrien Kerfourn, Clément Medrinal, Guillaume Prieur, Yann Combret, Jean-François Muir, Antoine Cuvelier, David Debeaumont, Gregory Reychler, Maxime Patout and Catherine Viacroze

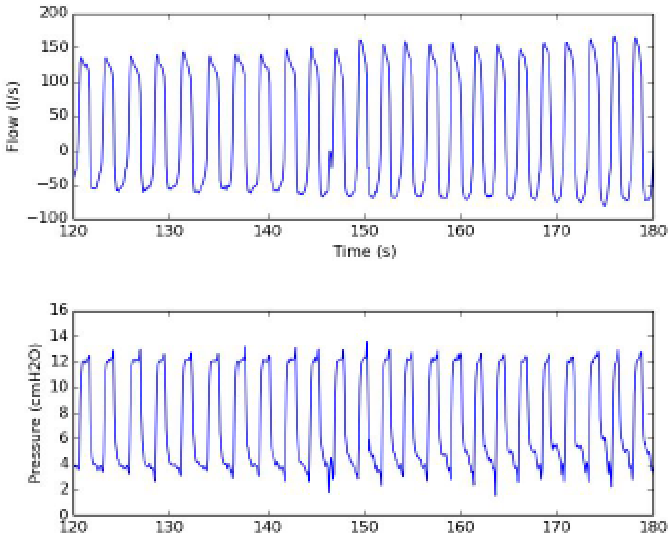

A

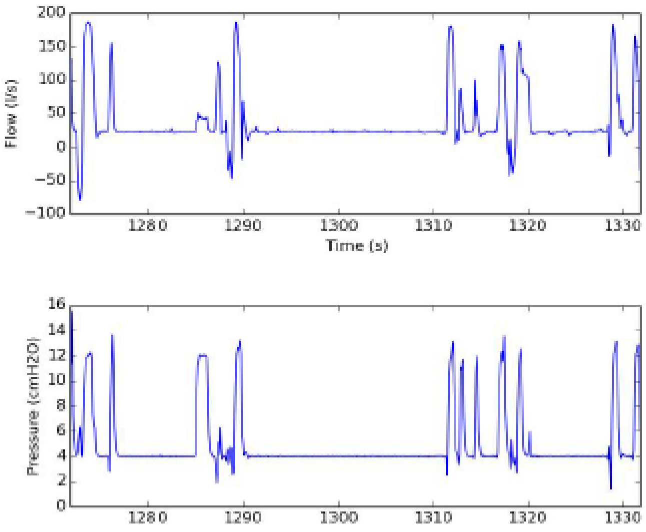

B

Figure S1. Comparison in ventilatory dynamic between the beginning (**A**) and the end (**B**) of exercise in a representative subject with nasal mask. The occurrence of ineffective effort at the end of exercise is in coherence with mouth leaks.

Table S1. Noninvasive ventilation settings.

Variables (Units)	Interface		Between-Group Comparison
	Oronasal Mask (*n* = 21)	Nasal Mask (*n* = 21)	*p*
Inspiratory support (cmH2O)	8 (8–10)	10 (8–10)	NS
Expiratory positive airway pressure (cmH2O)	4 (4–5)	4 (4–5)	NS
Slope	1 (1–3)	1 (1–2)	NS

Values expressed as medians (25th–75th percentile). NS, not significant.

Table S2. Comparison in flow and pressure between the beginning and the end of exercise.

Variable, (Units)	Moment During Exercise		Intra-Group Comparison
	First Minute	Last Minute	*p*
**Fini (L/min)**			
Oronasal interface, n = 17	33.2 (26.2–39.4)[b]	75.9 (44.6–109.2)[b]	$p < 0.01$
Nasal interface, n = 15	41.5 (22.2–49.0)[b]	38.0 (30.8–58.7)[b]	NS
Between-group comparison, p	NS	$p < 0.05$	
**Fmax (L/min)**			
Oronasal interface, n = 17	149.2 (124.7–174.7)[b]	214.0 (200.2–230.2)[b]	$p < 0.01$
Nasal interface, n = 15	128.1 (99.9–148.6)[b]	132.1 (110.5–179.0)[b]	$p < 0.01$
Between-group comparison, *p*	$p < 0.05$	$p < 0.01$	
**Pinspi (cmH2O)**			
Oronasal interface, n = 17	10.0 (9.3–11.8)[b]	8.7 (8.5–10.5)[b]	$p < 0.01$
Nasal interface, n = 15	10.3 (2.8)[a]	10.3 (2.4)[a]	NS
Between-group comparison, *p*	NS	NS	
**Pmax (cmH2O)**			
Oronasal interface, n = 17	13.9 (2.0)[a]	14.7 (2.1)[a]	$p < 0.01$
Nasal interface, n = 15	13.7 (3.1)[a]	14.3 (3.2)[a]	$p < 0.05$
Between-group comparison, *p*	NS	NS	
**Pmin (cmH2O)**			
Oronasal interface, n = 17	4.3 (0.6)[a]	3.3 (0.7)[a]	$p < 0.01$
Nasal interface, n = 15	4.1 (0.6)[a]	3.8 (0.5)[a]	$p < 0.05$
Between-group comparison, *p*	NS	$p < 0.05$	
**Pdiff (cmH2O)**			

Oronasal interface, n = 17	9.6 (2.1)[a]	11.4 (2.2)[a]	$p < 0.01$
Nasal interface, n = 15	9.6 (2.8)[a]	10.5 (2.9)[a]	$p < 0.01$
Between-group comparison, *p*	NS	NS	

[a]Values expressed as means (SD). [b]Values expressed as medians (25th–75th percentile). Fini: flow at the beginning of the cycle; Fmax: maximal flow; Pinspi: mean inspiratory pressure; Pmax: maximal pressure; Pmin: minimal pressure; Pdiff: differential pressure. NS, not significant.

Table S3. Comparison of flow and pressure between subjects with IE AI ≤ 10% or > 10% for nasal interface.

**Variables (Units)**	**Proportion of Ineffective Effort Asynchrony Index (%)**		**Between-Group Comparison**
	**≤10% (*n* = 8)**	**>10% (*n* = 7)**	***p***
$P_{inspi,beg}$ (cmH2O)	11.3 (9.7–14.4)	8.6 (8.2–9.2)	$p < 0.05$
$P_{inspi,end}$ (cmH2O)	10.3 (8.0–13.3)	9.1 (8.9–10.0)	NS
$P_{min,var}$ (cmH2O)	−0.47 (−0.6–−0.28)	0.00 (−0.12–0.01)	$p < 0.05$
IRR_{var}	7.4 (5.5–13.0)	3.4 (−1.8–5.3)	NS
Leaks (L/min)	8.2 (4.5–14.4)	25.6 (10.8–32.1)	$p < 0.05$
$F_{ini,beg}$ (L/min)	22.2 (19.3–23.2)	48.9 (46.2–51.8)	$p < 0.01$
$F_{ini,var}$ (L/min)	15.0 (9.3–43.3)	−10.5 (−12.9–2.6)	$p < 0.01$
$F_{ini,end}$ (L/min)	39.5 (29.3–58.1)	37.7 (32.9–48.9)	NS
$F_{max,beg}$ (L/min)	142.7 (132.2–148.9)	102.8 (91.4–118.5)	NS
$F_{max,end}$ (L/min)	179.0 (171.4–181.2)	110.5 (101.4–126.0)	$p < 0.01$
$F_{max,var}$ (L/min)	36.3 (31.5–45.2)	1.9 (−3.6–12.5)	$p < 0.05$
$F_{diff,beg}$ (L/min)	112.8 (106.5–126.3)	50.1 (42.9–78.0)	$p < 0.01$
$F_{diff,end}$ (L/min)	122.3 (104.8–143.2)	72.8 (61.8–74.8)	$p < 0.01$
τ_{beg} (ms)	447.4.8 (438.1–488.8)	425.0 (376.2–461.8)	NS
τ_{end} (ms)	448.2 (403.0–496.3)	432.7 (356.9–513.66)	NS

Values expressed as medians (25th–75th percentile). Pinspi: mean inspiratory pressure; beg: outcome assessed at the beginning of exercise; Pmin: minimal pressure; var: difference between the end minus the beginning of exercise; IRR: instantaneous respiratory rate; Fini,: flow at the beginning of the cycle; end: outcome assessed at the end of exercise; Fmax: maximal flow; Fdiff: differential flow; τ: pressure rise time. NS, not significant.

Table S4. Comparison of cycle time, instantaneous respiratory rate and Ti/Ttot between interfaces.

Variables (Units)	Interface		Between-Group Comparison
	**Oronasal Mask (*n* = 21)**	**Nasal Mask (*n* = 21)**	***p***
IRR_{mean}	25.5 (4.9)[a]	23.7 (4.6)[a]	$p < 0.05$
IRR_{var}	7.1 (5.2)[a]	4.3 (5.9)[a]	NS
CT_{mean}	2.2 (2.2–2.8)[b]	2.6 (2.3–2.9)[b]	$p < 0.05$
$Ti/Ttot_{mean}$ (%)	29.2 (0.1)[a]	29.3 (0.1)[a]	NS
τ_{mean} (ms)	458.8 (419.5–530.1)[b]	477.1 (448.7–497.7)[b]	NS

[a]Values expressed as mean (SD). [b]Values expressed as medians (25th–75th percentile). IRR: instantaneous respiratory rate; CT: cycle time; Ti/Ttot: ratio between inspiratory time and total time; τ: pressure rise time. NS, not significant.
